# The Olfactory System of *Dolichogenidea gelechiidivoris* (Marsh) (Hymenoptera: Braconidae), a Natural Enemy of *Tuta absoluta* (Meyrick) (Lepidoptera: Gelechiidae)

**DOI:** 10.3390/ijms26157312

**Published:** 2025-07-29

**Authors:** Shu-Yan Yan, He-Sen Yang, Cong Huang, Gui-Fen Zhang, Judit Arnó, Jana Collatz, Chuan-Ren Li, Fang-Hao Wan, Wan-Xue Liu, Yi-Bo Zhang

**Affiliations:** 1 Ministry of Agriculture and Rural Affairs Key Laboratory of Sustainable Crop Production in the Middle Reaches of the Yangtze River (Co-Construction by Ministry and Province), Hubei Engineering Technology Center for Forewarning and Management of Agricultural and Forestry Pests, College of Agriculture, Yangtze University, Jingzhou 434025, China; 2 State Key Laboratory for Biology of Plant Diseases and Insect Pests, Key Laboratory for Prevention and Management of Invasive Alien Species of Ministry of Agriculture and Rural Affairs, Institute of Plant Protection, Chinese Academy of Agricultural Sciences, Beijing 100193, China; 3 Sustainable Plant Protection Department, Institute for Research and Technology in Agriculture (IRTA), 08348 Cabrils, Barcelona, Spain; 4 Agroscope, Research Division Agroecology and Environment, Reckenholzstrasse 191, 8046 Zurich, Switzerland

**Keywords:** *Dolichogenidea gelechiidivoris*, Braconidae, antennal sensillum, microstructures, odorant-binding proteins (OBPs), odorant receptors (ORs)

## Abstract

The parasitoid wasp *Dolichogenidea gelechiidivoris* is a key koinobiont solitary endoparasitoid of the invasive agricultural pest *Tuta absoluta*. This study investigates both the morphological and molecular foundations of sex-specific olfactory differentiation in this species. Morphological analysis revealed that males possess significantly longer antennae (2880.8 ± 20.36 μm) than females (2137.23 ± 43.47 μm), demonstrating pronounced sexual dimorphism. Scanning electron microscopy identified similar sensilla types on both sexes, but differences existed in the length and diameter of specific sensilla. Transcriptomic analysis of adult antennae uncovered molecular differentiation, identifying 11 odorant-binding proteins (OBPs) and 20 odorant receptors (ORs), with 27 chemosensory genes upregulated in females and 4 enriched in males. Integrating morphological and molecular evidence demonstrates complementary sexual specialization in the olfactory apparatus of *D. gelechiidivoris*. Linking these findings to the potential functions of different sensilla types, as discussed in the context of prior research, provides crucial insights into the sex-specific use of volatile cues. These findings provide critical insights into the use of volatile signals in this highly relevant species for biological control targeting *T. absoluta*.

## 1. Introduction

Insects generally have a variety of sensilla on their antennae, which play a pivotal role in insect biology [1,2,3,4]. The sensillum is a specific region of the exoskeleton that contains formative cells, sensory nerve cells, and sometimes auxiliary cells [5]; the sensory cells receive various stimuli transmitted by the outer cuticular structure. Sensilla act as independent sensory units and include a number of different types, each with specific morphological features. The morphology of antennal sensilla of many insects has been systematically investigated. For example, Böhm bristles in *Anastatus disparis* are found only at the radicula and scape–pedicel junction, likely aiding in antennal movement or position sensing for accurate host signal detection [6]. Sensilla basiconica and sensilla trichodea are mainly involved in the function of olfactory recognition, and can sense the sex pheromones of insects and the volatiles released by plants [7,8]. Sensilla chaetica is often regarded as a non-chemical receptor that is primarily responsible for sensing mechanical stimuli [9]; they are also present in other species and are considered to be gustatory organs with roles in the host location process [10,11]. Sensilla placodea in females is used to detect plant volatiles early in the search for oviposition hosts, while in males, it may help locate mates [12]. Studies indicate that sensilla campaniformia function as humidity and temperature detectors, while sensilla coeloconica mediate temperature sensing and likely thermohygroreception [13].

Parasitoids are insects (wasps, flies, or beetles) in which adult females forage for hosts, usually other insects, and deposit their eggs in, on, or near these hosts [14]. The host then provides the food resource for the developing parasitoid larvae. Parasitoids are interesting biological models for addressing questions in olfaction and behavioral ecology because the foraging behavior of females is directly linked to their reproductive success. Furthermore, since males and females rely on different cues for their reproductive success, i.e., host and conspecific cues, respectively, parasitoids provide ideal study organisms to investigate sex-specific differentiation in olfactory systems. The evolutionary success of these parasitoids fundamentally relies on their sophisticated chemoreception system, where antennae are important sensory organs for insects to perceive their complex external environment and to transmit intraspecific and interspecific information [15,16]. Interspecific volatiles from hosts and their food plants play a crucial role for host location, whereas non-volatile kairomones mediate host recognition and host acceptance [17]. Intraspecific volatile pheromones, on their own or together with kairomones, and non-volatile signals such as cuticular hydrocarbons mediate mating.

This intricate chemoreception ecology is supported by the molecular and neural differentiation of antennal sensilla. These sensory organs are equipped with various types of receptors that perceive environmental stimuli, converting chemical signals into electrical impulses transmitted through neuronal networks, thereby coordinating adaptive behavioral responses [18,19,20]. The chemical receptors on antennal sensilla operate through a sophisticated molecular cascade: Volatile odorants are initially captured by odorant-binding proteins (OBPs) and chemosensory proteins (CSPs), the latter of which may facilitate transport of non-volatile ligands or act as co-receptors, which shuttle hydrophobic molecules through the aqueous receptor lymph. This transport facilitates ligand interaction with transmembrane olfactory receptors (ORs) anchored on dendritic membranes of sensory neurons [21,22]. The OR complex is further modulated by sensory neuron membrane proteins (SNMPs), which stabilize OR–ligand binding and enhance signal fidelity [23]. The resulting receptor activation triggers ion flux via SNMP-associated channels, effectively transducing chemical information into graded potentials [21,24]. Post stimulation, odorant-degrading enzymes (ODEs) rapidly inactivate odorants to maintain sensory neuron sensitivity and temporal resolution of signal processing [21]. These signals undergo central integration in higher brain centers, such as the antennal lobe, ultimately governing context-specific behaviors. The neuronal integration, therefore, allows for the processing of information such as the recognition of complex odor blends [25].

**Figure 1:**
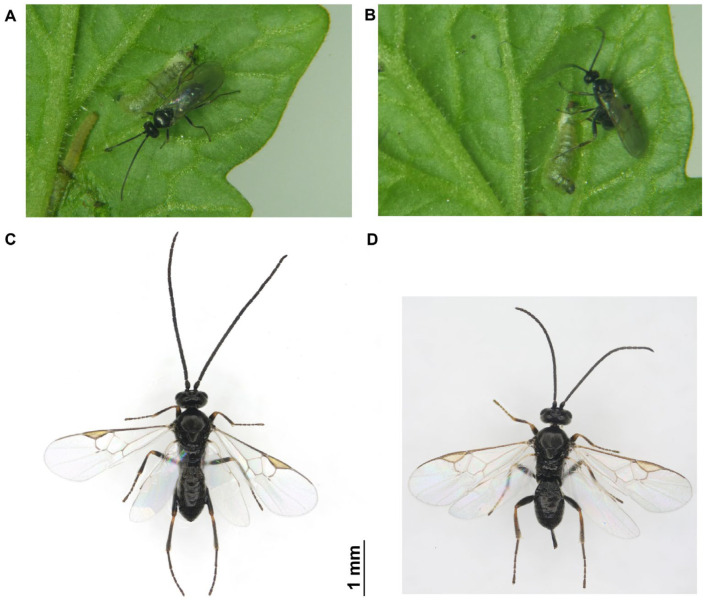
*D. gelechiidivoris* adults and parasitism behavior on *T. absoluta* larvae. (**A**) Female parasitic wasp searching for an oviposition site on a *T. absoluta* larva. (**B**) Female wasp parasitizing a *T. absoluta* larva. (**C**) Morphology of a male adult. (**D**) Morphology of a female adult.

The South American tomato leafminer, *Tuta absoluta* (Meyrick) (Syn.: *Phthorimaea absoluta*) (Lepidoptera: Gelechiidae), is a globally invasive and highly destructive pest of solanaceous crops [26,27]. Following its initial invasion of the Xinjiang Uygur Autonomous Region in August 2017, *T. absoluta* is now established in large parts of China, posing a significant threat to the tomato production industry [28]. *Dolichogenidea gelechiidivoris* (Marsh 1975) (Syn.: *Apanteles gelechiidivoris*) (Hymenoptera: Braconidae: Microgastrinae), originating from South America, is a koinobiont solitary larval endoparasitoid that targets *T. absoluta*, capable of parasitizing all larval instars but showing a preference for the first to second instar larvae (Figure 1A–D) [29,30]. It has been extensively employed in classical biocontrol programs against *T. absoluta* in East African [29,31,32] and also recorded as accidentally introduced in Europe and Algeria [33,34]. Since both *T. absoluta* and *D. gelechiidivoris* originated in South America, they have undergone a prolonged period of co-evolution. To further investigate the unique chemical communication mechanisms between parasitoids and its hosts, studying the olfactory system of *D. gelechiidivoris* holds substantial scientific significance.

Although recent ultrastructural investigations on braconid antennal morphology have established a framework linking sensilla architecture to ecological functions [35], the antennal sensory systems of *D. gelechiidivoris* remain unexplored. This study addresses two critical dimensions: (1) elucidating how structural specialization of antennal sensilla correlates with ecological adaptations in *D. gelechiidivoris* and (2) deciphering the molecular basis of sex-specific chemosensory behaviors through differential gene expression analysis. The resulting insights will advance our understanding of Hymenopteran chemical ecology while using a species of high biological control significance as a model.

## 2. Results

### 2.1. General Description of Antennae

**Figure 2:**
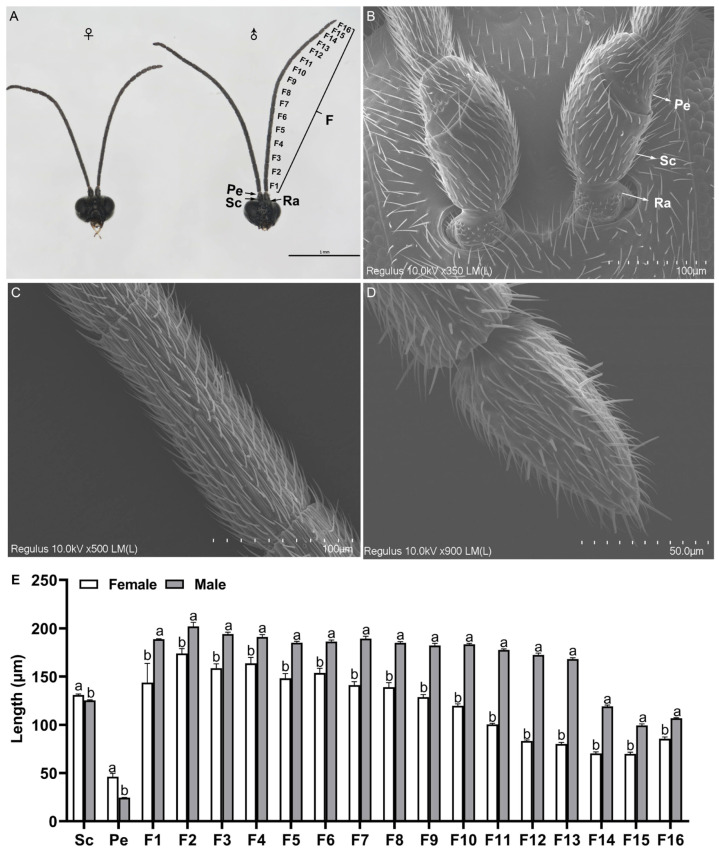
Morphological characteristics of the antennae of female and male adults of *D. gelechiidivoris* and comparative measures. (**A**) Antenna of female and male showing the radicle (Ra), scape (Sc), pedicel (Pe), flagellum (F). Scanning electron micrograph of (**B**) scape and pedicel, (**C**) flagellum subsegment, and (**D**) tip of antenna excised from a *D. gelechiidivoris* antenna. (**E**) Lengths of sampled female and male (Sc–F16) antennomeres. Different lowercase letters above bars indicate significant differences between males and females (independent sample *t*-test, *p* < 0.05).

Both male and female antennae of *D. gelechiidivoris* consist of four segments: radicle (Ra), scape (Sc), pedicel (Pe), and flagellum (F). The radicle is a short cylindrical segment that is inserted into the antenna socket. The scape is relatively elongated and robust, exhibiting a cylindrical shape with slight swelling in the middle (Figure 2B–D). The pedicel is cone-shaped and notably shorter than the scape. The flagellum, as the terminal segment of the antennae, comprises 16 subsegments (F1–F16) (Figure 2A). The antennae of *D. gelechiidivoris* exhibit sexual dimorphism. Specifically, the total length of the male antenna was 2880.8 ± 20.36 µm, which was significantly longer than that of the female antenna, measuring 2137.23 ± 43.47 µm (Table S2, *t* = −14.402, df = 10, *p* < 0.001). Additionally, there were significant differences in the width of each antennae segment between the two sexes (Figure 2E, Table S1).

### 2.2. Scanning Electron Microscopy of the Adult Antennae

#### 2.2.1. Different Types and Morphology of Sensilla with Distribution

**Figure 3:**
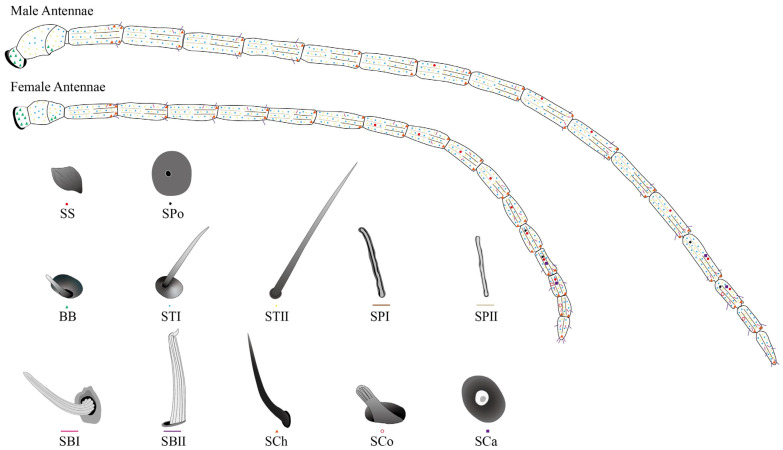
Schematic diagram of antennal sensilla morphology and distribution in male and female *D. gelechiidivoris*.

Sensillar diversity and sexual dimorphism were investigated in both male and female antennae. Twelve distinct types of sensilla were identified across both sexes (Figure 3), including Böhm bristles (BBs), two subtypes of sensilla trichodea (ST-I, ST-II), two subtypes of sensilla placodea (SP-I, SP-II), two subtypes of sensilla basiconica (SB-I, SB-II), sensilla chaetica (SCh), sensilla coeloconica (SCo), sensilla campaniformia (SCa), sensilla squamous (SS), and smell pores (SPo). Figure 3 details the characteristic morphology and antennal distribution patterns of each sensillum type. Sexual dimorphism was evident not only in overall antennal length (Figure 3, Table S1), but also in the dimensions of individual sensilla (Figure 3). Furthermore, significant differences were observed in sensillar distribution density. Typically, sensilla were more densely distributed on male antennae compared to females, irrespective of sensillum type.

#### 2.2.2. Böhm Bristles (BBs)

**Figure 4:**
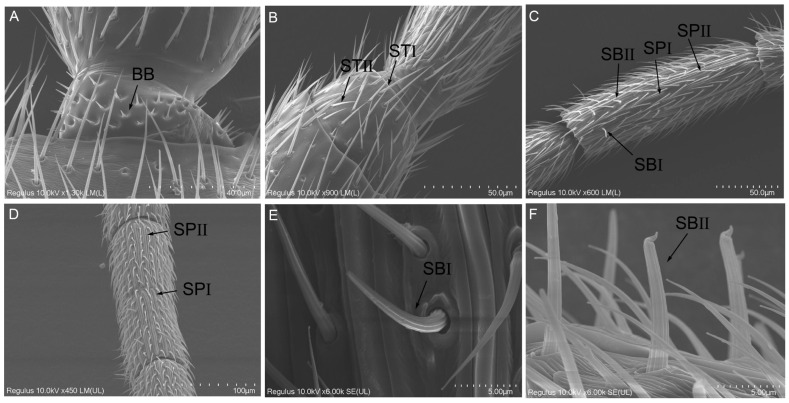
Morphological characteristics of some antennal sensilla of *D. gelechiidivoris* female and male adults. (**A**) Böhm’s bristles (BB), (**B**) sensilla trichodea I (STI) and sensilla trichodea II (STII), (**C**) sensilla placodea I (SPI), sensilla placodea II (SPII), sensilla basiconica I (SBI), and sensilla basiconica II (SBII), (**D**) sensilla placodea I (SPI) and sensilla placodea II (SPII), (**E**) sensilla basiconica I (SBI), and (**F**) sensilla basiconica II (SBII).

The BBs are fixed on the radicula bases and pedicel bases (Figure 4A) in both males and females. These bristles emerge from the concave regions on the antennae surface, extending nearly perpendicular to it. Characterized by their short and pointed morphology, the BBs gradually taper from the base to the tip. No pores were detected on the surface of the BBs. In females, the BBs measured 3.49 ± 0.08 μm in length with a basal diameter of 1.28 ± 0.02 μm, whereas in males, they were 4.50 ± 0.10 μm long with a basal diameter of 1.43 ± 0.02 μm. A significant difference was observed between the sexes in terms of the average length (*t =* −7.285, df = 10, *p* < 0.001) and basal diameter (*t =* −6.358, df = 10, *p* < 0.001) of the BBs (Table S2).

#### 2.2.3. Sensilla Trichodea (ST)

ST are distributed extensively across all regions of the antennae in both sexes, except the radicula, and represented the most numerous sensillum type. ST can be classified into two subtypes, STI and STII, based on differences in socket morphology and length. STI exhibits a hair-like structure with a slender end and a sharp tip that curves slightly toward the segment apex (Figure 4B). These structures are anchored into a slightly elevated concave socket within the cuticular surface, and longitudinal ridges are observed on their surfaces. In females, STI measured 43.60 ± 1.57 μm in length with a basal diameter of 1.75 ± 0.02 μm, while in males, they measured 26.12 ± 0.67 μm in length with a basal diameter of 1.84 ± 0.02 μm (Table S2). STII share a similar appearance to STI but are shorter in length and more predominantly located at the apex of antennal pedicel and flagellum. STII are slightly curved hairs with smooth surfaces and minimal pores, tapering to a fine sharp tip. STII lack a socket structure and are only slightly elevated above the cuticle (Figure 4B). In females, STII measured 25.4 ± 0.33 μm in length with a basal diameter of 1.45 ± 0.01 μm, while in males, they measured 18.87 ± 0.48 μm in length with a basal diameter of 1.38 ± 0.03 μm. No significant difference was observed in the basal diameter of ST between the sexes; however, the length of ST in female wasps was significantly greater than that in male wasps (STI, *t* = 9.372, df = 6.77, *p* < 0.001; STII, *t* = 10.273, df = 10, *p* < 0.001) (Table S2).

#### 2.2.4. Sensilla Placodea (SP)

SP are plate-like sensory organs of *D. gelechiidivoris* characterized by a central surface resembling a roof ridge. These structures were tightly adhered to the antenna surface and surrounded by elevated cuticular structures. SP are exclusively distributed on the flagellum of the antenna (Figure 4C,D). Based on their morphology, SP can be categorized into SPI and SPII. SPI were characterized by a central elevation shaped like a roof ridge, with the bluntly rounded ends that are slightly wider and a middle portion that is slightly narrower. The orientation of SPI is nearly parallel to the longitudinal axis of the antenna, and they are distributed in alternating rows at the distal end of the flagellomere. The average length of SPI in male adults is significantly greater than that in female adults (Table S2, *t =* −19.662, df = 10, *p* < 0.001), although no significant difference was observed in width (Table S2, *t =* −2.192, df = 6.636, *p* = 0.067). SPII exhibit a plate-like structure in the center, which is slightly recessed below the antenna surface, with both ends protruding beyond the antenna surface. Similar to SPI, the average length of SPII in male adults is significantly greater than in female adults (Table S2, *t =* −4.883, df = 10, *p* = 0.001), but no significant difference in width was detected between males and females (Table S2, *t =* −0.689, df = 10, *p* = 0.506).

#### 2.2.5. Sensilla Basiconica (SB)

SB of *D. gelechiidivoris* are characterized by their greater thickness compared to ST, tapering gradually from the base to the tip. The base of these sensilla is cup-shaped, while the tip is blunt and arch-shaped with longitudinal grooves on the surface (Figure 4C). Based on their length and apical morphology, SB can be categorized into two types: SBI and SBII. SBI exhibit a gradual curvature in the middle and lower parts, tapering to a blunt apex. These cone-shaped sensilla taper progressively from the base to the tip, with a distinct curvature in the middle and lower sections, resulting in a rounded blunt tip (Figure 4E). Their unique shape makes them more easily distinguishable from other sensilla. Compared to ST, SBI are more perpendicular to the antennal axis and extend significantly above the plane of other sensilla. These sensilla are distributed in the middle of each antennal flagellomere in both male and female adults. Notably, the length of SBI in male adults was significantly greater than that in female adults (Table S2, *t* = −4.883, df = 10, *p* < 0.001), while basal diameter was not significantly different between sexes (Table S2, *t* = −0.689, df = 10, *p* = 0.506). SBII exhibit a slightly thicker base that is embedded into a depression in the cuticle, with tips specialized into a cap-like structure that curves backward. These sensilla are fewer in number and scattered, with an increasing density towards the end of each antennal flagellomere (Figure 4F). In male adults, the length (Table S2, *t* = −10.674, df = 10, *p* < 0.001) and basal diameter (Table S2, *t* = −12.134, df = 10, *p* < 0.001) of SBII in male adults were significantly larger than those in female adults.

#### 2.2.6. Sensilla Chaetica (SCh)

**Figure 5:**
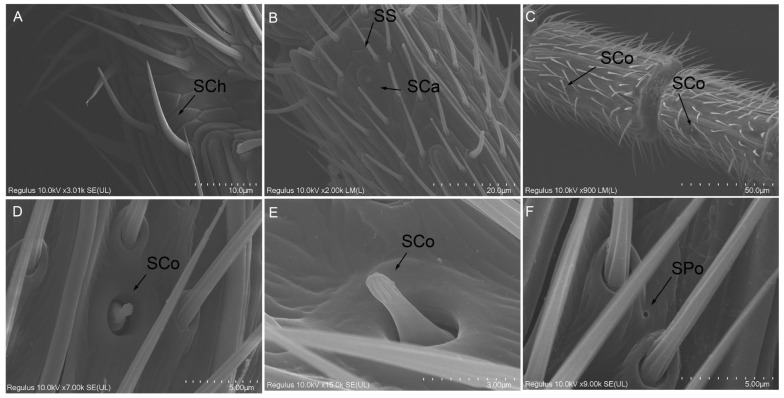
Morphological characteristics of some antennal sensilla of *D. gelechiidivoris* female and male adults: (**A**) sensilla chaetica (SCh), (**B**) sensilla squamous (SS) and sensilla campaniformia (SCa), (**C**–**E**) sensilla coeloconica (SCo), and (**F**) smell pores (SPo).

SCh are exclusively localized at the apical end of each flagellomere and exhibit a lower density compared to other types of sensilla. SCh resemble spines and are characterized by their shorter length and greater rigidity relative to STI. They possess a swollen base, blunt tip, and smooth surface, and are embedded into a concave cuticle at their base (Figure 5A). Notably, the length (Table S2, *t* = −4.261, df = 5.373, *p* = 0.007) and basal diameter (Table S2, *t* = −6.667, df = 10, *p* < 0.001) of SCh in male adults were significantly larger than those in female adults.

#### 2.2.7. Sensilla Coeloconica (SCo)

In contrast to all previously described sensilla, each peg-shaped SCo originates from a deep, smooth-walled, and circular cavity on the antennal surfaces of both sexes of *D. gelechiidivoris*. These structures feature a central papillate protrusion surrounded by petal-like margins (Figure 5C). The margins curve toward the cavity center, with their tips interlocking and converging. Additionally, these margins display distinct fine longitudinal ridges oriented upward (Figure 5D,E). The base diameter (Table S2, *t* = −8.997, df = 10, *p* < 0.001) and height (Table S2, *t* = −24.515, df = 10, *p* < 0.001) of SCo were significantly larger in male adults compared to female adults (Table S2).

#### 2.2.8. Sensilla Campaniformia (SCa)

SCa are characterized by a dome-shaped flexible cuticular cap surrounded by a concentric groove, which is slightly elevated above the exoskeleton. The major and minor axes of the flat elliptical base in the SCa sensor of female adults were significantly larger than those of male adults (Table S2, *t* = 6.412, df = 10, *p* < 0.001, *t* = 5.439, df = 10, *p* < 0.001), whereas the diameter of the central concentric groove structure was significantly smaller in females compared to males (Table S2, *t* = −4.239, df = 10, *p* = 0.002).

#### 2.2.9. Sensilla Squamous (SS)

SS, characterized by longitudinal textures on its surface, taper from the base to the tip, forming a leaf-like shape that becomes progressively thinner and narrower (Figure 5B). These sensilla are scattered across the subsegments of the flagellum, located between the hairy sensilla in the middle and distal regions of each subsegment. The length of SS in male adults was significantly greater than that in female adults (Table S2, *t* = −5.293, df = 10, *p* < 0.001), while the width in male adults was significantly smaller than that in female adults (Table S2, *t* = 5.729, df = 10, *p* < 0.001).

#### 2.2.10. Smell Pores (SPo)

SPo are generally small in size and dispersed across the surface, typically located near the base of the sensory hairs at their attachment point to the antennal surface (Figure 5F).

### 2.3. Transcriptome-Based Identification of Differentially Expressed DoliOBPs and DoliORs

**Figure 6:**
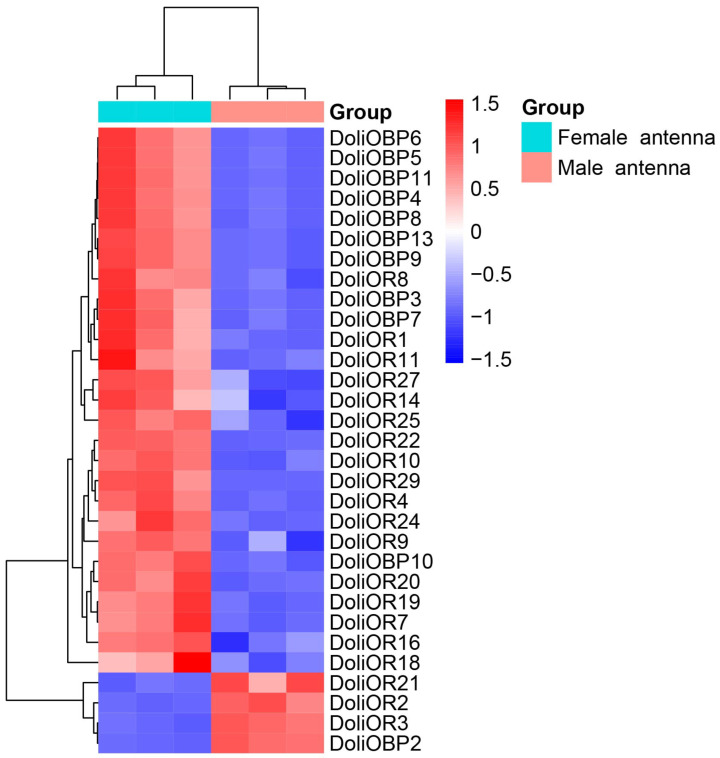
Heatmap of odorant-binding protein gene (OBP) and olfactory receptor gene (OR) expression levels between female and male antennae. The data were visualized using a color-coded scale ranging from blue (low expression) to red (high expression), with values normalized between −1.5 and 1.5. Hierarchical clustering was performed to group samples and genes based on expression patterns, as indicated by the dendrograms at the top and left side of the heatmap.

Based on antennal transcriptome data from *D. gelechiidivoris*, this study systematically investigated the sex-specific expression patterns of two pivotal chemosensory gene families: odorant-binding proteins (OBPs) and olfactory receptors (ORs). Analysis of chemosensory genes revealed 11 OBP genes (84.6% of the 13 identified OBP genes) and 20 OR genes (64.5% of the 31 identified OR genes) with pronounced sex-biased expression (Figure 6, Tables S3 and S4). Hierarchical clustering and heatmap analyses revealed distinct sex-specific expression signatures in antennal tissues (Figure 6). Notably, the clustering analysis demonstrated a clear separation between the male and female groups. Specifically, 27 genes (*DoliOBP3*, *DoliOBP4*, *DoliOBP5*, *DoliOBP6*, *DoliOBP7*, *DoliOBP8*, *DoliOBP9*, *DoliOBP10*, *DoliOBP11*, *DoliOBP13*, *DoliOR1*, *DoliOR4*, *DoliOR7*, *DoliOR8*, *DoliOR9*, *DoliOR10*, *DoliOR11*, *DoliOR14*, *DoliOR16*, *DoliOR18*, *DoliOR19*, *DoliOR20*, *DoliOR22*, *DoliOR24*, *DoliOR25*, *DoliOR27*, and *DoliOR29*) exhibited significantly female-biased upregulation, while 4 genes (*DoliOBP2*, *DoliOR2*, *DoliOBP3*, and *DoliOR21*) showed male-specific enrichment.

## 3. Discussion

The parasitoid wasp *D. gelechiidivoris* is commonly employed as a biological control agent against the major lepidopteran invasive pests *T. absoluta*. However, the mechanisms underlying *D. gelechiidivoris* host localization remain inadequately understood, thereby constraining its optimization as an efficient and effective biological control agent. The discovery of sexual dimorphism in the olfactory system of *D. gelechiidivoris* offers critical insights into the evolutionary adaptations of parasitoids and their ecological interactions with host species, including *T. absoluta*.

First, the adult antennae of *D. gelechiidivoris* were examined using SEM due to their role as the primary olfactory organs in insects. This study provides the first comprehensive morphological characterization of antennal sensilla in this species, identifying 12 distinct types of sensilla with significant differences in size and type between males and females. A clear sexual dimorphism is evident, with males exhibiting a bead-like structure along the flagellar segments, while females display a shorter bead-like structure at the distal segments. Furthermore, a significant difference in antennal length between the sexes was observed. This structural variation is consistent with patterns observed in other wasps, such as *Baryscapus dioryctriae* [36], *Macrocentrus cingulum* [37], and *Microplitis pallidipes* [35], suggesting conserved adaptations to sex-specific ecological roles. Males, which are responsible for locating mates, likely benefit from elongated antennae that enhance pheromone detection efficiency by increasing the sensory surface area [38]. Conversely, females prioritize the detection of olfactory cues for host location during oviposition [39].

The functional specialization of sensilla further elucidates behavioral divergence. Böhm bristles (BBs), localized at the scape–pedicel junction, act as proprioceptors responsible for sensing the position and movement direction of the antennae [40]. For instance, in *Sitodiplosis mosellana* and *Sirex noctilio*, BBs are hypothesized to detect gravity and buffer external mechanical stimuli, thereby controlling antenna movement [41,42]. In *D. gelechiidivoris*, BBs are exclusively found at the scape–pedicel junction, suggesting their role in sensing antennal position relative to the body and aiding in orientation adjustment during flight. ST represent a prominent component of the antennal sensory system in Hymenoptera, characterized by their widespread distribution, numerical dominance, and extensive surface area. These sensilla are categorized into porous and non-porous subtypes based on ultrastructural features: the former facilitates chemoreception by enabling odorant diffusion through wall pores into the sensillar lymph, while the latter primarily mediates mechanosensation by responding to tactile stimuli, airflow variation, or postural adjustment [36,43]. In *D. gelechiidivoris*, both sexes exhibit non-porous ST with longitudinal grooves consistent with previous morphological descriptions [44,45], indicating their specialization in mechanical perception [36].

SP, distinguished by their dense neural innervation, are pivotal for chemosensory processes (olfaction/gustation) in insects [44,46]. Studies on *Microplitis croceipes* revealed that type I SP mediate long-range mate detection in males, while type II assist females in host localization [47]. In *D. gelechiidivoris*, dimorphism in size between type I and II SP suggests analogous functional specialization, potentially mirroring the behavioral ecology of those parasitoids.

SB in *D. gelechiidivoris* are classified into two subtypes (SBI and SBII): type I exhibits an erect morphology with blunt tips, while type II features a specialized cap-like apex curved posteriorly, consistent with observations in *S. noctilio* [42]. Previous studies suggest that thick-walled type I SB detect mechanical stimuli, while thin-walled type II with apical caps may be involved in chemoreception [48]. These structural similarities suggest conserved functional roles in *D. gelechiidivoris*. SCh, identified as tactile mechanoreceptors, assist wasps in assessing the position of their antennae relative to the environment [49]. For instance, *S. mosellana* utilizes these sensilla for mechanosensation [41]. In *D. gelechiidivoris*, SCh are structurally similar to those previously described and are distributed around the scape and pedicel in both males and females, with females exhibiting significantly greater length and basal diameter. The smooth non-porous surface and morphological congruence with *S. mosellana* strongly suggest homologous mechanosensory functions.

Notably, the SCo, which resemble hygroreceptive or thermoreceptive sensilla in other parasitoids [42], likely play a role in microclimate assessment during oviposition. In contrast, SCa are critical for evaluating host suitability by detecting humidity, CO_2_, and thermal gradients [48,50,51]. The distribution and function of these sensilla vary among insect species. Typically, those located on the pedicel are proprioceptive mechanoreceptors sensitive to environmental changes in temperature and humidity, whereas those on the flagellum serve as gustatory receptors, but are also responsive to environmental changes [52]. In this study, the SCa of *D. gelechiidivoris* were exclusively found on the flagellum, suggesting their involvement in taste perception and the detection of temperature and humidity. The SS, localized at the basal flagellomeres in both sexes, have been documented as significant mechanoreceptors potentially involved in wind direction perception and environmental interaction across various insects [53]. In *D. gelechiidivoris*, we speculate that they may function as mechanoreceptors or anemotactic sensors. The SPo, hypothesized as chemoreceptors sensitive to pheromones [41,53], are interspersed among ST and SP in *D. gelechiidivoris*. Although their distribution has been documented in *Aphidius gifuensis* [54], further investigation is required to validate their functional roles.

Furthermore, the successful completion of the antennal transcriptome sequencing of *D. gelechiidivoris* adults represents a crucial advancement in elucidating its olfactory system and exploring its ecological application potential for pest control. Chemosensory genes, including odorant-binding proteins (OBPs), chemosensory proteins (CSPs), and chemoreceptors such as odorant receptors (ORs), ionotropic receptors (IRs), and gustatory receptors (GRs), have been extensively identified in Hymenopteran species [55], particularly within the Braconidae family [56]. Based on the antennal transcriptome data of *D. gelechiidivoris*, this study identified transcripts encoding 13 OBPs and 31 ORs. Notably, the number of OBPs identified is closely comparable to the 20 OBPs previously characterized in *Cotesia vestalis* [57]. In contrast, the OR family exhibits significant expansion in *D. gelechiidivoris* compared to the 25 ORs identified in *Microplitis mediator* [58]. This substantial expansion suggests an evolutionary adaptation in the olfactory recognition capabilities of *D. gelechiidivoris*, likely linked to its host-searching strategy, reflecting a need for finer discrimination within complex chemical environments and potentially a broader host search range. Specifically, our analysis revealed that 27 chemosensory genes were predominantly highly expressed in female antennae, indicating their direct involvement in female-specific olfactory-driven behaviors and likely association with the host parasitization process [57]. Conversely, four chemosensory genes displayed male-specific expression, suggesting their participation in male-specific pheromone detection or search behaviors [59]. This sexually dimorphic expression pattern demonstrates the differential shaping of the olfactory system in males and females by natural selection: the expansion of female-associated genes serves ecological niche adaptation for host searching, while the specialization of male-associated genes focuses on optimizing reproductive efficiency.

Ubiquitously expressed *DoliORs* and *DoliOBPs* may facilitate general odorant perception. These results are consistent with previous reports of sexual dimorphism in chemosensory gene expression across diverse insect species [60,61,62,63], indicating that differences in olfactory requirements between the sexes, driven by divergent behaviors such as host plant volatile perception, oviposition site selection, and predator avoidance, likely underlie molecular and morphological adaptations. Future research should focus on functionally validating candidate genes using RNA interference (RNAi) or CRISPR/Cas9 editing alongside the electrophysiological characterization of sensilla subtypes. Furthermore, comparative genomic analyses of chemosensory gene families within the Braconidae family could elucidate the evolutionary trajectories of these adaptations.

## 4. Materials and Methods

### 4.1. Insects

Adults of *D. gelechiidivoris* used in this study originated from a laboratory colony established with specimens collected from *T. absoluta* larvae in Matarió, Barcelona, Spain (41.5445° N, 2.4470° E), in 2022. The *T. absoluta* host population for parasitic wasps was obtained from samples of infected tomatoes collected from fields in Yuxi City, Yunnan Province, China, and reared in the laboratory for more than 10 consecutive generations. All insects were maintained in mesh cages (60 cm × 60 cm × 40 cm, 100 mesh) at 26 ± 1 °C, 60% ± 5% RH, and a photoperiod of 16:8 (L:D) h and supplied with cotton soaked in honey water (20% *v*/*v*) placed on the top of the cage.

### 4.2. Scanning Electron Microscopy

Newly emerged adult wasps of both sexes (0–24 h post eclosion) were collected and immediately anesthetized by placing them on ice. Next, we removed the antennae from their heads under a Stemi 508 stereoscope (Zeiss, Oberkochen, Germany) and cleaned them in an ultrasonic bath for 30 s. Their antennae were subsequently immersed in 2.5% glutaraldehyde at 4 °C for 24 h. Following fixation, it was rinsed three times with phosphate buffer solution (0.1 M PBS, pH 7.2). The specimens were then dehydrated in a graded alcohol series (75%, 80%, 85%, 90%, and 100%) for 15 min each, followed by two 10 min acetone washes for further drying. Then, the antennae specimens were air-dried for 24 h. Subsequently, the antennae were mounted on metal stubs using double-sided adhesive tape and coated with gold through sputtering coating. Gold was removed from solid electrodes by ion bombardment under high vacuum using an EM ACE600 coater (Leica, Wetzlar, Germany). Following mounting and sputtering coating, the gold-coated insect antennal sensilla were observed on the stage of a Leica scanning electron microscope (Thermo Fisher Scientific, Waltham, MA, USA) using a 10 kV accelerating voltage. A total of 12 adult antennae per sex were used for measurement. We recorded images onto a computer and used SEM particle size statistics software version 1.5 to measure the length and basal width of each sensillum.

### 4.3. RNA Isolation

The antennae of newly emerged adult wasps of both sexes (0–24 h after eclosion) were dissected under the stereo microscope (Leica M205C, Wetzlar, Germany). Antennae from 50 males and 50 females were collected separately and pooled to form three biological replicates for each sex. Freshly excised antennae were immediately immersed in RNAwait stabilization solution (Solarbio, Beijing, China) and flash-frozen in liquid nitrogen. Total RNA extraction was conducted using the TaKaRa MiniBEST Universal RNA Extraction Kit (Takara Bio, Kusatsu, Japan), with an on-column DNase I treatment step included to ensure complete removal of genomic DNA contamination. The integrity of the extracted RNA was assessed using the Agilent 2100 TapeStation System (Agilent Technologies, Santa Clara, CA, USA), with all samples exhibiting RNA Integrity Numbers (RIN) ≥ 8.0. RNA purity and concentration were further validated using a NanoDrop^TM^ One/OneC spectrophotometer (Thermo Fisher Scientific, USA), confirming absorbance ratios (A260/A280 and A260/A230) > 1.8. Qualified RNA samples were stored at −80 °C for subsequent transcriptome library construction.

### 4.4. RNA-Seq and Transcriptome Assembly

Raw sequencing data obtained from the Illumina XPlus platform (Illumina, CA, USA) were subjected to rigorous quality control procedures. Adapter-containing reads were removed and low-quality sequences were filtered using Trimmomatic v0.39 with the following criteria: (1) reads containing ambiguous bases (N) exceeding 10% of total length; (2) reads with ≥50% of bases having Phred quality scores ≤ 10; and (3) minimum length threshold set to 50 bp, with shorter reads being discarded. Cleaned data were then assembled into transcripts using Trinity v2.15.1. The coding potential of Unigenes was predicted using CPC2 v1.0 and putative coding sequences were annotated by aligning to the NCBI non-redundant protein database (Nr) via BLAST+ v2.12.0 (E-value < 1 × 10^−5^), enabling the identification of novel genes.

### 4.5. Differential Gene Expression Analysis

The Trinity-assembled transcriptome was used as the reference sequence and clean reads from each sample were aligned to this reference using HISAT2 v2.2.1. Transcript abundances were quantified as FPKM via StringTie v2.1.3b. Differential expression analysis was conducted with DESeq2 v1.30.1, with applied thresholds of |log_2_(fold change)| ≥ 1 and adjusted *p*.adj < 0.01. Significantly differentially expressed olfactory-related genes were functionally annotated and visualized using hierarchical clustering heatmaps (R 4.1.2 pheatmap package) based on z-score-normalized FPKM values. Heatmap was plotted by https://www.bioinformatics.com.cn (last accessed on 10 December 2024), an online platform for data analysis and visualization.

### 4.6. Statistical Analysis

Based on Schneider (1964) [64] and Zacharuk (1980) [65] classification system, and with reference to previously documented studies on Hymenopteran sensilla [36,66,67,68,69], this study conducted the classification and identification of the sensilla. The lengths and widths of antennal scapes, pedicels, and flagella were subsequently measured. Data visualization was performed using GraphPad Prism 8 (GraphPad Software, SanDiego, CA, USA). Independent sample *t*-tests (GraphPad Prism 8) were conducted to compare the dimensions of each antennal segment and measurements of various sensillum types between males and females. Asterisks indicate significant differences (*, *p* < 0.05). Statistical results were reported as means ± standard error (SE).

## Data Availability

All of the data that support the findings of this study are available in the main text.

## Supplementary Materials

The supporting information can be downloaded at https://www.mdpi.com/article/10.3390/ijms26157312/s1.
