# Supplementary material for: The Olfactory System of Dolichogenidea gelechiidivoris (Marsh) (Hymenoptera: Braconidae), a Natural Enemy of Tuta absoluta (Meyrick) (Lepidoptera: Gelechiidae)
Source: Int J Mol Sci. 2025 Jul 29;26(15):7312. doi: 10.3390/ijms26157312 (PMC12347633; doi:10.3390/ijms26157312)
Supplement: Supplementary file 1 [file ijms-26-07312-s001.zip › ijms-3713926-supplementary/ijms-3713926-Supplementary File.pdf]

Appendix S

Table S1. Morphological differences in antennae between female and male adults of *D. gelechiidivoris*.

Antennae	Number of sections	Female/μm	Male/μm
Scape	1	131.00±0.94	125.33±0.56
Pedicel	1	46.05±3.35	24.42±0.24
Flagellum	F1	143.67±18.34	188.75±0.47
	F2	174.00±4.62	202.00±3.98
	F3	158.67±4.22	194.17±1.74
	F4	163.67±5.72	191.17±2.19
	F5	148.17±4.62	185.17±1.30
	F6	153.83±4.31	186.33±1.48
	F7	141.17±3.31	189.50±2.16
	F8	139.00±4.27	185.00±1.13
	F9	128.67±2.50	182.17±2.10
	F10	119.83±1.75	183.50±0.84
	F11	100.47±1.02	177.50±1.22
	F12	83.27±0.96	172.50±1.71
	F13	80.05±1.55	168.17±1.26
	F14	70.35±1.43	119.00±1.39
	F15	69.88±1.70	99.30±1.54
	F16	85.50±1.74	106.83±0.55
Full length	18	2137.23±43.47	2880.80±20.36

Note: Data in the table were presented as the mean ± SE and calculated from measured values of 12 male and female adults, respectively.

Table S2. Size of antennal sensilla of *D. gelechiidivoris* adults.

Type of Sensilla	Length/μm		Basal diameter/μm		Width/μm		Ring Width/μm		height/μm		Sensilla outer wall
	Female	Male	Female	Male	Female	Male	Female	Male	Female	Male	
Böhm bristles	3.49±0.08*	4.50±0.10	1.28±0.02*	1.43±0.02	-	-	-	-	-	-	Smooth
Sensilla trichodea I	43.60±1.57*	26.12±0.67	1.75±0.02	1.84±0.02	-	-	-	-	-	-	longistudinal lines
Sensilla trichodea II	25.40±0.33*	18.87±0.48	1.45±0.01	1.38±0.03	-	-	-	-	-	-	longistudinal lines
Sensilla placodea I	77.10±0.72*	95.83±0.49	-	-	2.05±0.01	2.14±0.03	-	-	-	-	smooth
Sensilla placodea II	69.73±1.04*	81.25±1.88	-	-	2.78±0.03	2.84±0.07	-	-	-	-	smooth
Sensilla basiconica I	8.10±0.34*	12.65±0.19	1.67±0.02*	2.07±0.02	-	-	-	-	-	-	longistudinal lines
Sensilla basiconica II	5.63±0.08*	7.56±0.41	1.26±0.01*	1.43±0.02	-	-	-	-	-	-	longistudinal lines
Sensilla chaetica	19.35±0.42	18.95±0.47	1.70±0.02	1.64±0.01	-	-	-	-	-	-	smooth
Sensilla campaniformia	8.55±0.09	7.60±0.10	-	-	5.23±0.05	4.75±0.06	0.57±0.02	0.66±0.01	-	-	fine longitudinal ridges on top
Sensilla coeloconica	-	-	2.80±0.02	3.09±0.02	-	-	-	-	1.28±0.01	1.7±0.01	patterned on top
Sensilla squamous	5.74±0.04	-	-	-	3.42±0.08	-	-	-	-	-	smooth

Note: Data are presented as the mean ± SE. For a given segment and measurement, means followed by asterisk differ significantly between males and females (*t*-test, $P<0.05$). '–' indicates absence.

Table S3. *D. gelechiidivoris* assembled Unigenes with annotation as candidate odorant-binding proteins of *D. gelechiidivoris* adults.

Transcript ID	Gene name	Length (aa)	Predicted Function	BLASTX best hit	Identify (%)	FA-VS-MA _log2FC	FA-VS-MA _regulated	padj
g2781_i15	DoliOBP1	170	odorant-binding protein 1	ABM05968.2 [*Microplitis mediator*]	73.88	-0.97	normal	2.50E-10
g5300_i2	DoliOBP2	156	PREDICTED: general odorant-binding protein 83a-like	XP_014296295.1 [*Microplitis mediator*]	51.91	-5.26	down	1.45E-17
g14904_i0	DoliOBP3	152	odorant-binding protein 3	ABM05970.1 [*Microplitis mediator*]	56.92	2.42	up	2.82E-29
g15190_i0	DoliOBP4	150	odorant-binding protein 3	ABM05970.1 [*Microplitis mediator*]	56.92	2.40	up	1.63E-38
g18213_i0	DoliOBP5	150	odorant-binding protein 3	ABM05970.1 [*Microplitis mediator*]	56.92	2.42	up	4.95E-37
g16894_i0	DoliOBP6	149	odorant-binding protein 3	ABM05970.1 [*Microplitis mediator*]	55.81	2.41	up	3.60E-40
g15189_i0	DoliOBP7	148	odorant-binding protein 3	ABM05970.1 [*Microplitis mediator*]	56.92	2.44	up	4.64E-26
g17221_i0	DoliOBP8	148	odorant-binding protein 3	ABM05970.1 [*Microplitis mediator*]	56.92	2.45	up	9.23E-35
g1313_i14	DoliOBP9	148	odorant-binding protein 4	ABM05971.2 [*Microplitis mediator*]	50.35	2.94	up	1.09E-45
g2781_i10	DoliOBP10	147	PREDICTED: general odorant-binding protein 83a-like	XP_008543434.1 [*Microplitis demolitor*]	76.03	2.26	up	1.14E-36
g19958_i0	DoliOBP11	141	odorant-binding protein 3	ABM05970.1 [*Microplitis mediator*]	57.85	2.44	up	9.41E-40
g194_i60	DoliOBP12	135	PREDICTED: general odorant-binding protein 56d	XP_008548249.1 [*Microplitis demolitor*]	55.64	0.88	normal	2.79E-06
g9151_i0	DoliOBP13	130	odorant-binding protein 4	ABM05971.2 [*Microplitis mediator*]	50.00	2.82	up	6.11E-45

Table S4. *D. gelechiidivoris* assembled Unigenes with annotation as candidate olfactory receptors.

Transcript ID	Gene name	Length (aa)	Predicted Function	BLASTX best hit	Identify (%)	FA-VS-MA _log2FC	FA-VS-MA _regulated	padj
g6885_i0	DoliOR1	455	PREDICTED: putative odorant receptor 92a	XP_008555850.1 [*Microplitis demolitor*]	62.73	1.28	up	4.20E-10
g543_i2	DoliOR2	434	PREDICTED: odorant receptor 83a-like	XP_008555847.1 [*Microplitis demolitor*]	50.23	-4.10	down	5.14E-44
g3778_i1	DoliOR3	434	PREDICTED: odorant receptor 82a-like	XP_008555986.1 [*Microplitis demolitor*]	62.97	-5.02	down	1.46E-05
g1548_i5	DoliOR4	433	PREDICTED: odorant receptor 83a-like	XP_008555847.1 [*Microplitis demolitor*]	79.81	5.25	up	4.68E-09
g834_i1	DoliOR5	426	PREDICTED: odorant receptor 4-like	XP_008543412.1 [*Microplitis demolitor*]	76.07	0.97	normal	6.34E-06
g243_i3	DoliOR6	419	PREDICTED: odorant receptor Or2-like	XP_014297486.1 [*Microplitis demolitor*]	81.88	0.74	normal	3.13E-03
g187_i14	DoliOR7	419	odorant receptor 21	AKO89985.1 [*Microplitis mediator*]	53.05	1.75	up	2.27E-34
g7518_i0	DoliOR8	416	odorant receptor 48	AKO90012.1 [*Microplitis mediator*]	76.57	1.72	up	6.86E-15
g6863_i0	DoliOR9	414	PREDICTED: putative odorant receptor 92a	XP_008555850.1 [*Microplitis demolitor*]	67.8	2.13	up	3.59E-04
g4520_i2	DoliOR10	409	PREDICTED: odorant receptor 13a	XP_008552621.1 [*Microplitis demolitor*]	78.37	1.86	up	5.52E-12
g8571_i1	DoliOR11	405	odorant receptor 34	AKO89998.1 [*Microplitis mediator*]	79.24	1.59	up	5.75E-13

g3569_i0	DoliOR12	400	odorant receptor	AZQ24891.1 [*Aphidius gifuensis*]	60.45	0.68	normal	5.02E-03
g3260_i4	DoliOR13	399	odorant receptor 22	AKO89986.1 [*Microplitis mediator*]	55.53	0.79	normal	1.11E-03
g8333_i0	DoliOR14	398	PREDICTED: odorant receptor 13a-like	XP_014298633.1 [*Microplitis demolitor*]	73.87	1.24	up	4.37E-03
g882_i1	DoliOR15	395	PREDICTED: odorant receptor 4-like	XP_014298181.1 [*Microplitis demolitor*]	76.73	0.95	normal	3.87E-03
g6710_i4	DoliOR16	393	PREDICTED: odorant receptor Or1-like	XP_014297040.1 [*Microplitis demolitor*]	64.08	1.41	up	1.83E-08
g368_i18	DoliOR17	393	PREDICTED: odorant receptor 33a-like	XP_008559709.1 [*Microplitis demolitor*]	77.53	0.86	normal	1.02E-03
g468_i4	DoliOR18	392	PREDICTED: odorant receptor 13a-like	XP_014298546.1 [*Microplitis demolitor*]	69.77	1.36	up	5.38E-03
g8838_i1	DoliOR19	389	PREDICTED: odorant receptor 85b-like, partial	XP_008556117.1 [*Microplitis demolitor*]	79.13	1.28	up	2.62E-09
g3144_i0	DoliOR20	384	PREDICTED: putative odorant receptor 71a	XP_014297039.1 [*Microplitis demolitor*]	79.54	1.14	up	1.42E-27
g6535_i1	DoliOR21	380	PREDICTED: odorant receptor 46a, isoform A-like	XP_014295857.1 [*Microplitis demolitor*]	57.89	-1.32	down	4.43E-04
g2620_i0	DoliOR22	377	PREDICTED: odorant receptor 49b-like	XP_008546952.1 [*Microplitis demolitor*]	56.45	2.31	up	1.45E-35
g1451_i0	DoliOR23	377	odorant receptor 41	AKO90005.1 [*Microplitis mediator*]	77.48	0.90	normal	4.23E-03
g1857_i0	DoliOR24	373	PREDICTED: odorant receptor 13a-like	XP_014298179.1 [*Microplitis demolitor*]	63.28	1.84	up	9.36E-28

g9575_i0	DoliOR25	315	PREDICTED: odorant receptor 13a-like	XP_014298633.1 [*Microplitis demolitor*]	68.26	1.26	up	1.15E-03
g8982_i0	DoliOR26	276	PREDICTED: odorant receptor 4-like	XP_014295941.1 [*Microplitis demolitor*]	76.85	0.78	normal	5.51E-07
g7952_i0	DoliOR27	276	PREDICTED: odorant receptor 13a-like	XP_014298633.1 [*Microplitis demolitor*]	66.67	1.17	up	2.37E-03
g7823_i1	DoliOR28	260	PREDICTED: odorant receptor 22c-like	XP_008560943.1 [*Microplitis demolitor*]	50.38	-0.62	normal	1.31E-03
g4827_i1	DoliOR29	143	PREDICTED: putative odorant receptor 85d	XP_008553862.1 [*Microplitis demolitor*]	90.85	3.35	up	5.78E-51
g19332_i0	DoliOR30	140	PREDICTED: odorant receptor 4-like	XP_014295941.1 [*Microplitis demolitor*]	92.81	0.80	normal	3.21E-04
g15385_i0	DoliOR31	140	PREDICTED: odorant receptor 4-like	XP_014295941.1 [*Microplitis demolitor*]	92.03	0.87	normal	9.50E-04
